# The Best Illuminant for Small Hospitals

**Published:** 1911-09-09

**Authors:** C. Hoddle


*To the Editor of* The Hospital.

### THE BEST ILLUMINANT FOR SMALL HOSPITALS.

*To the Editor of* The Hospital.

Sir,—Referring to your recent article under the title of "The Artificial Illumination of the Small Hospital," may we point out that this article contains a rather remarkable error, which error is calculated to injure the acetylene industry very considerably? The present price of carbide of calcium for the manufacture of acetylene is not £20 per ton, but £13 5s., and allowing an average of 15s. per ton for carriage from the depôt, this brings the total cost to £14 per ton. There are about thirty depôts scattered over Great Britain, so that the above carriage allowance is a liberal one. It is about ten years ago (in the early stage of the industry) since carbide cost £20 per ton. In our opinion, for hospital lighting acetylene is quite as economical as petroleum, and we would add that, from a hygienic point of view, it is quite the best illuminant for any purpose whatever.

We are, yours faithfully,

(Thorn and Hoddle Acetylene Co., Ltd.),

C. Hoddle.

[We gladly publish the accompanying letter from the Thorn and Hoddle Acetylene Co., Ltd. The table given in the article referred to (May 20, 1911) was described as an approximate one, and £20 per ton was mentioned as the basis on which the figure was arrived at. The letter will awaken interest in acetylene as an economical illuminant for the small hospital, and, as pointed out in the article, quite a desirable one.—Ed.]

